# Recent Advances in Phthalocyanine and Porphyrin-Based Materials as Active Layers for Nitric Oxide Chemical Sensors

**DOI:** 10.3390/s22030895

**Published:** 2022-01-24

**Authors:** Darya Klyamer, Roman Shutilov, Tamara Basova

**Affiliations:** Nikolaev Institute of Inorganic Chemistry SB RAS, 3 Lavrentiev Pr., 630090 Novosibirsk, Russia; klyamer@niic.nsc.ru (D.K.); shutilov@niic.nsc.ru (R.S.)

**Keywords:** chemical sensors, chemiresistive sensors, electrochemical sensors, phthalocyanines, porphyrins, nitric oxide

## Abstract

Nitric oxide (NO) is a highly reactive toxic gas that forms as an intermediate compound during the oxidation of ammonia and is used for the manufacture of hydroxylamine in the chemical industry. Moreover, NO is a signaling molecule in many physiological and pathological processes in mammals, as well as a biomarker indicating the course of inflammatory processes in the respiratory tract. For this reason, the detection of NO both in the gas phase and in the aqueous media is an important task. This review analyzes the state of research over the past ten years in the field of applications of phthalocyanines, porphyrins and their hybrid materials as active layers of chemical sensors for the detection of NO, with a primary focus on chemiresistive and electrochemical ones. The first part of the review is devoted to the study of phthalocyanines and porphyrins, as well as their hybrids for the NO detection in aqueous solutions and biological media. The second part presents an analysis of works describing the latest achievements in the field of studied materials as active layers of sensors for the determination of gaseous NO. It is expected that this review will further increase the interest of researchers who are engaged in the current level of evaluation and selection of modern materials for use in the chemical sensing of nitric oxide.

## 1. Introduction

Nitric oxide (NO) is a colorless highly reactive toxic gas that forms as an intermediate compound during the oxidation of ammonia to nitric acid and is used for the manufacture of hydroxylamine in the chemical industry [1,2]. Exhaled NO can react quickly with oxygen in the lungs to form nitrogen dioxide, which is a strong lung irritant. On the other hand, nitric oxide is a signaling molecule in many physiological and pathological processes in mammals, including blood pressure regulation, immune response and neural communication [3,4,5], and the 1998 Nobel Prize in Physiology or Medicine was awarded for the discovery of its role as a signaling molecule of the cardiovascular system [6]. According to the Web of Science database, the growing interest in the detection of nitric oxide can be easily noticed: More than 3500 research and review articles have been published in the last ten years, and the number of publications continues to grow. 

An increase in the NO concentration of more than 10 ppb indicates a course of inflammatory processes in the respiratory tract [7,8,9,10,11]. It has been shown that an increase in the concentration of fractional exhaled nitric oxide (FENO) indicated some diseases of the respiratory tract, such as asthma [7,8,12,13], allergy [9,14,15] and chronic cough [10,16]. Moreover, in connection with the COVID-19 pandemics, scientists have begun to publish works in which the analysis of the nitric oxide concentration could be useful for the diagnostics of coronavirus disease [17,18]. Several sensor devices have already been produced for the detection of FENO [19]. NIOX-MINO (Aerocrine AB, Solna, Sweden) is one of the examples of such a new portable device based on electrochemical sensors used in clinical practice in Western Europe [20].

Recent studies [21,22,23] emphasize the importance of determining not only NO, but also its metabolites (nitrites, nitrates, 3-nitrotyrosine) that accumulate in biological fluids. For this reason, accurate detection and quantification of NO in both the gas phase and aqua media are important tasks for understanding the development of the disease. Several methods were used to measure NO, including Griess test [24,25], chemiluminescence [26], electron spin resonance (ESR) [27,28], fluorescent probe [29,30] and electrochemical sensing [31,32]. Apart from these, waveguide microwave sensors are also utilized for the detection of nitrogen oxides due to their simplicity, low cost, passive and non-contact operations [33,34]. The determination of NO in biological samples imposes a number of special requirements on sensor devices, such as low price, simple sample preparation, small size and a low detection limit of about 10 ppb.

A common requirement for sensor materials is their high sensitivity to the analyte at very low concentrations and selectivity in the presence of environmental factors. Semiconductors such as oxides [35,36], transition metal nitrides [37], carbon-containing materials [38,39], metal phthalocyanines (MPc) and porphyrins (MPor) [40,41,42] are widely used as sensor materials. The latter are popular materials because of their ability to change their resistive, electrochemical and electrocatalytic properties in a wide range, to vary their structure (viz. terminal substituents in ligands and the central metal) widely [43], as well as because of the high thermal and chemical stability of porpyrinoids compared to most other molecular materials. The advantages of MPc-based sensors also include their short response time, reversibility of the sensor response at room temperature and the possibility of obtaining films on flexible substrates [40,44]. It is known that MPc sensors are used to detect H_2_, NH_3_ and H_2_S gases, which are biomarkers of such diseases as malabsorption, kidney failure and halitosis [43,44,45]. The high catalytic activity of phthalocyanine and porphyrin complexes can be attributed to the unsaturated low-coordination environment of central metal ions coordinated with four isoindole subunits [46]. Their catalytic activity in the process of NO oxidation is reflected in most cases by a negative shift of the oxidation voltammetric feature of NO by ca. 0.15 V and by the two–three-fold increase in the current intensity compared to conventional bare electrodes [32,47].

In 2015, Dang et al. [48] published a review on the application of various nanomaterials for the detection of NO in physiological media, e.g., carbon nanomaterials, metal nanoparticles, semiconductor metal oxides and their nanocomposites. The review published by Bedioui et al. [32] reported studies on the detection of NO gas in biological systems using electrochemical sensors until 2010. In that study, Bedioui discussed basic principles of using electrochemical nickel porphyrin and Nafion sensor for detecting nitric oxide. In 2010, Nyokong et al. [49] presented a review on the use of MPc-based molecular materials as catalysts for electrochemical reactions. However, among many electrochemical reactions, their catalytic activity in the electrochemical oxidation of nitric oxide and nitrite has only been briefly described. The review of Goshi and co-workers was devoted to the development of various methods for determining NO in vivo and in vitro, including optical methods, chemiluminescence and some others [50]. 

**Figure 1:**
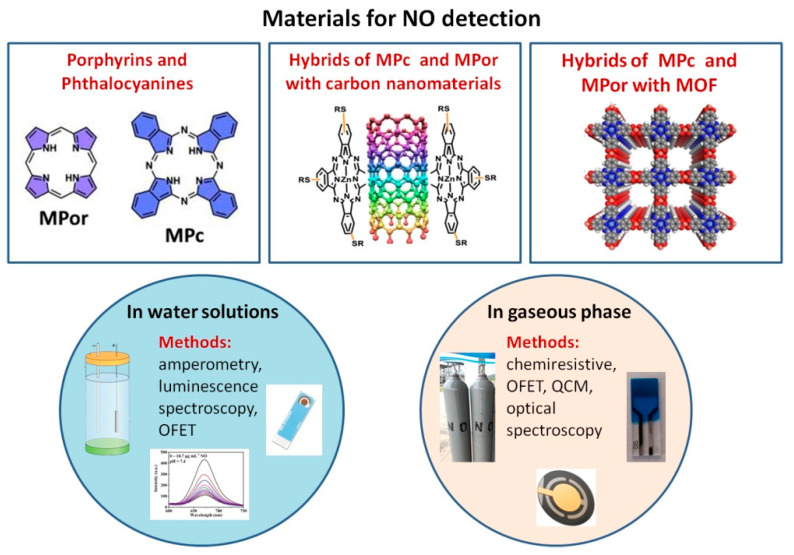
An overview of the main phthalocyanine- and porphyrins-based materials, which are used as active layers of chemical sensors for the detection of nitric oxide, discussed in this review.

This review analyzes the state of research over the past ten years in the field of applications of metal phthalocyanines, porphyrins and their hybrid materials as active layers of chemical sensors for the detection of nitric oxide (Figure 1), with a primary focus on chemiresistive and electrochemical ones. The first part of the review is devoted to the study of phthalocyanines and porphyrins, as well as their hybrids with carbon nanomaterials and metal-organic frameworks (MOF) for the detection of NO in aqueous solutions and biological media. The second part of the review presents an analysis of works describing the latest achievements in the field of materials based on phthalocyanines and porphyrins as active layers of chemiresistive and other sensors for the determination of gaseous NO.

## 2. Sensing Layers for Detecting NO in Aqueous Media

NO is an unstable, highly lipophilic, free radical molecule which has poor solubility in water but relatively higher solubility in lipid membranes [51]. The solubility of NO in phosphate buffered saline (PBS) solution is 1.8 mM at room temperature [52]. The half-life of NO in biological tissues is observed to be only three–five and about 500 s in pure aqueous solutions [21]. This imposes strict requirements both for ensuring the speed of the sensors being developed and for the preparation of analyzed solutions containing NO and its metabolites. When developing sensors for the direct determination of NO, the following methods of the preparation of nitric oxide in aqueous media are most often used [53,54,55]. Saturated NO solutions are obtained by bubbling NO gas through a deoxygenated PBS solution for an hour before saturation. Some researchers use sodium or potassium nitrite as a precursor to produce NO due to their disproportionation reaction in acidic solution (pH < 4) [55,56]. The resulting NO gas is passed through NaOH/KOH solutions to remove oxygen and other nitrogen oxides. Another way to produce nitric oxide is the reaction of nitric acid with copper. In some works, S-nitroso-N-acetyl-DL-penicillamine (SNAP) is used as a source of nitric oxide [57,58]. SNAP decomposes thermally or under the influence of ultraviolet light to form NO.

Electrochemical sensors offer the advantage of low detection limit, fast response, easy manufacturing and small size, allowing real-time and fast measurement of NO concentration in water media and biological samples [32,59]. A classical three-electrode configuration consisting of a working electrode, reference electrode and counter electrode is usually employed to detect NO. The electrochemical sensing of NO is based on the electrooxidation of NO to NO^+^ at a sensor electrode in a one electron process, followed by a homogeneous reaction to form nitrite (NO_2_^−^) [49]:

NO = NO^+^ + e^−^ first electrochemical reaction (1)

NO^+^ + OH^−^ = NO_2_^−^ + H^+^ (HNO_2_) second electrochemical reaction. (2)

NO_2_^−^ is electrochemically active and may undergo subsequent electrochemical oxidation at the sensor electrode to nitrate according to the following:

NO_2_^−^ + H_2_O → NO_3_^−^ + 2H^+^ + e^−^ third electrochemical reaction. (3)

The last reaction proceeds at a similar electrochemical potential as NO oxidation [60]. The recorded signal when NO is detected by the electrochemical method is a current generated on the electrode surface as a result of NO oxidation. The amount of oxidized NO is proportional to the current flowing between the electrodes in the electrochemical cell and the initial concentration of nitric oxide. 

The amperometric method is the most common technique employed for the measurement of NO concentration. Nevertheless, other techniques, such as differential pulse voltammetry (DPV), differential normal pulse voltammetry (DNPV), linear scanning voltammetry (LSV) and fast scan voltammetry (FSV), were also used to measure NO concentration. Analysis of the literature shows that most of the studies related to the development of NO sensors are devoted to the search for new materials for electrode modification. This is necessary to increase the selectivity and decrease the detection limit of the electrochemical sensor material. Porphyrin or phthalocyanine derivatives and their composite or hybrid materials are widely used to modify electrodes for selective detection of NO.

### 2.1. Porphyrins and Phthalocyanines 

The application of metal porphyrins and phthalocyanines as active sensing layers in chemiresistive, optical, quartz crystal microbalance (QCM) and electrochemical sensors are widely described in the literature [61,62,63]. This interest is related to the ability of porphyrins and phthalocyanines to form complexes with most metal ions, thereby providing rich and diverse coordination chemistry. MPc and MPor have extended π-systems which allow them to undergo fast redox processes and thereby facilitate electron transfer to a variety of molecules [49]. It is known that these complexes have a catalytic effect in various redox processes due to the displacement of voltammetric characteristics in the direction of reducing overvoltage and enhancing current responses [32].

Literature analysis shows that phthalocyanines containing redox active metal centers are mainly used as electron mediators in hybrid systems, e.g., phthalocyanines of cobalt, copper, manganese and iron. The reaction scheme for NO electrocatalytic oxidation on metal porphyrins and phthalocyanines was studied in a number of works [64,65,66]. The proposed mechanism of NO oxidation on an electrode modified by MPc (or MPor) considered the fact that NO was first adsorbed on the MPc and partially increased the electron density with subsequent electron transfer to form NO^+^:

PcM(II) ⇔ [PcM(III)]^+^ + e^−^ (4)

[PcM(III)]^+^ + NO ⇔ [PcM(III)^δ−^(NO)^δ+^]^+^ (5)

[PcM(III)^δ−^(NO)^δ+^]^+^ ⇔ [PcFe(III)]^+^ + NO^+^+ e^−^ (6)

After that, NO^+^ could further react with water with the formation of nitrite (NO2^−^) through a homogeneous reaction. In some cases, the formed electrochemically active NO2^−^ was converted to nitrate (NO3^−^) by a two-electron process [67]:

NO^+^ + H_2_O → HNO_2_ + H^+^ (7)

HNO_2_ ⇔ NO2^−^ + H^+^ (8)

NO_2_^−^ + H_2_O → NO_3_^−^ + 2H^+^ + 2e^−^ (9)

**Figure 2:**
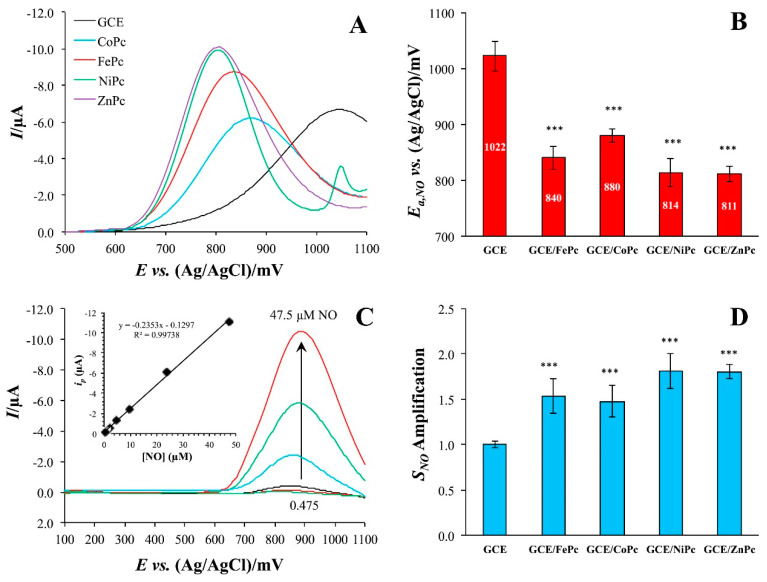
(**A**) Differential pulse voltammograms of bare and MPc-modified GC electrodes in the presence of 23.75 μM NO in pH 7.4 PBS with (**B**) corresponding peak potentials. (**C**) Overlay of DPV traces collected in the presence of different NO concentrations on a FePc-modified GCE (Inset: calibration curve from the peak currents as a function of concentration). (**D**) Nitric oxide sensitivity amplification of MPc-modified electrodes relative to the bare GCE. *** = *p* < 0.001 with respect to GCE. Reprinted with permission from Ref. [47]. Copyright 2018 Elsevier.

It has been reported that the introduction of different central metals can affect the coordination site of NO [68], the orientation of bonds [69], the electronic structure [70] and the oxidation potential [71]. For example, Brown and Schoenfisch [47] investigated the effect of central metal on the catalytic properties of MPc (M = Fe(II), Co(II), Ni(II) and Zn(II)) by DPV method. For this purpose, the MPcs were deposited onto glassy carbon electrodes (GCE) by drop-casting their solutions in pyridine. The modification of electrodes by MPcs caused a noticeable anodic peak shift towards lower potentials of about 200 V (Figure 2A). The NO peak was shifted in the order ZnPc ~ NiPc > FePc > CoPc as shown in Figure 2B. Apart from this, modification of the electrode with all investigated phthalocyanines led to an increase in the sensitivity of the sensor to NO by 1.5 times. Although the type of the central metal did not have a significant effect on the degree of amplification, the same order was maintained as when the peak was shifted. The linear dependence of the peak current on NO concentration was observed below 50 μM (Figure 2C). It is important to note that when investigating the possibility of NO detection in the presence of nitrite, among the studied MPcs only FePc demonstrated a noticeable difference between experimental and theoretical selectivity to nitrite, which indicated the most specific catalytic enhancement. 

**Figure 3:**
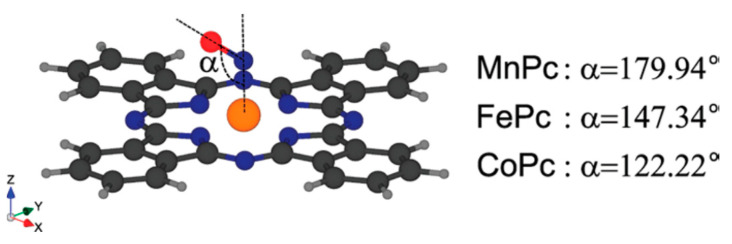
The most stable configurations for NO adsorbed on MPc after full optimization. Reprinted with permission from [69] Copyright 2010, American Chemical Society.

In an attempt to explain the influence of the central metal on the interaction between MPc or MPor and NO molecules, the authors of other works turned to quantum chemical calculations [68,69,72]. For example, Nguyen with co-authors [68,69] studied the adsorption of nitric oxide (NO) on various MPcs with M = Mn, Fe, Co, Ni, Cu, Zn using DFT calculations. The most stable configuration for NO adsorbed on MPc, obtained as a result of full optimization, is shown in Figure 3. The authors concluded that the orientation of NO relative to the MPc macrocycle was strongly dependent on the *d*-electron state near the Fermi level in different metal phthalocyanines.

The binding energies of NO with MPc in these structures were −1.738, −1.899 and −1.552 eV for MnPc-NO, FePc-NO and CoPc-NO species, respectively. The fact that FePc and CoPc strongly bind NO may also explain the higher oxidation potential of NO compared to NiPc and ZnPc (Figure 2B). The bonds were shown to form by the hybridization of π* orbital of NO molecule and two types of *d* orbitals of metals (*d_π_* and *d_z_*^2^). This led to the opening of the HOMO-LUMO gap. The binding energies of NO with MPc with M = Ni, Cu and Zn were much lower and equal to −0.103, −0.032 and −0.005 eV. For this reason, the interaction of NO with these MPcs was classified as physisorption and their electronic structure was kept intact. 

The literature analysis shows that phthalocyanines and porphyrins of Co(II) and Fe(II) are most often used for the modification of sensing electrodes. The examples of phthalocyanine and porphyrin-based sensors used for the detection of NO in aqueous solutions and biological media are summarized in Table 1. 

**Figure 4:**
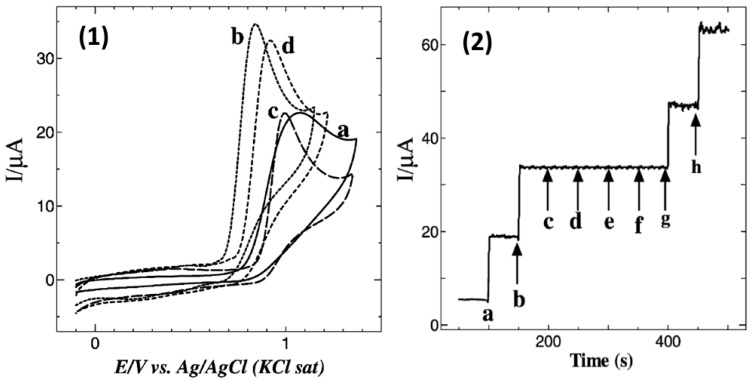
(**1**) CVs obtained for 0.5 mM NO at (a) bare GC electrode, (b) 4α-Co^II^TAPc, (c) 4α-H_2_TAPc and (d) 4α-Co^II^TAPc modified GC electrodes in 0.2 M PB solution (pH 2.5) at a scan rate of 0.05 V/s. (**2**) Amperometric I(T) curve for the addition of 10 μM of NO (a and b) and 1 mM each of glucose (c), urea (d), oxalate (e) and NaCl (f) and final additions of 10 μM of NO (g,h) at 4α-Co^II^TAPc SAM modified GC electrode in 0.2 M PBS (pH 2.5). Reprinted with permission from Ref. [56]. Copyright 2010 John Wiley and Sons.

Apart from the drop-casting technique, the method of immersion of electrodes in MPc or MPor solutions was also utilized for electrode modification. 1,8,15,22-tetraaminophthalocyanatocobalt(II) (4α-Co^II^TAPc) was used for the modification of GCE to improve its sensitivity to NO [56]. Self-assembly of 4α-Co^II^TAPc on GCE was achieved by its immersion in a solution of the phthalocyanine (1 mM) in DMF for 3 h with the following sonication in pure DMF to remove the rest of the non-adsorbed molecules. The self-assembly of porphyrin and phthalocyanine molecules on the surface of GCEs was achieved due to π-π interaction between their aromatic macrocycles and graphite sheets. GCE modified by 4α-Co^II^TAPc demonstrated a peak corresponding to NO oxidation at 0.84 V with a higher oxidation current than in the case of bare GCE (Figure 4(1)) and GCE modified by 4α-H_2_TAPc. The authors noted that the nitrogen atmosphere was maintained throughout the scan to avoid the interfering effects of nitrites, although it could not be excluded that oxidation of NO_2_^−^ to NO_3_^−^ did not occur under those conditions. At the same time, the authors did not mention the interfering effect of nitrites in the article. 

The calculated detection limit was found to be 1.4 × 10^−10^ M, while the linear range was from 3 × 10^−9^ to 30 × 10^−9^ M. The enhanced sensor response to NO was explained by the electrocatalytic activity of 4α-Co^II^TAPc with the involvement of the central metal. The site of oxidation is an important factor in the electrocatalytic activity of MPc complexes. Redox properties are observed at the central metal in MnPc, FePc and CoPc while ring-based processes occur in ZnPc, NiPc and CuPc. MPc complexes with metal-based oxidation process are expected to show better electrocatalytic activity than ring-based MPc complexes. Catalytic reactions involving CoPc are thus normally mediated by the redox reactions centered in the central metal [71]. The electrode could be used for the detection of NO in the presence of high concentrations of glucose, urea, oxalate, NaCl (up to several mM), ascorbic acid and dopamine (20-fold excess) (Figure 4(2)) and demonstrated quite good recovery for NO in human blood serum samples. 

The electrodeposition and electropolymerization techniques are also widely used to modify the electrode surface. Ryo Matsuoka et al. [58] prepared a sensor based on iron tetrakis(3-thienyl) porphyrin (FeT3ThP) for the determination of O^−^ and NO species, which were deposited onto a plastic formed carbon (PFC) by electropolymerization. S-nitroso-N-acetyl-DL-penicillamine (SNAP) was used for the NO generation. The porphyrin layer was covered with a Nafion film.

**Figure 5:**
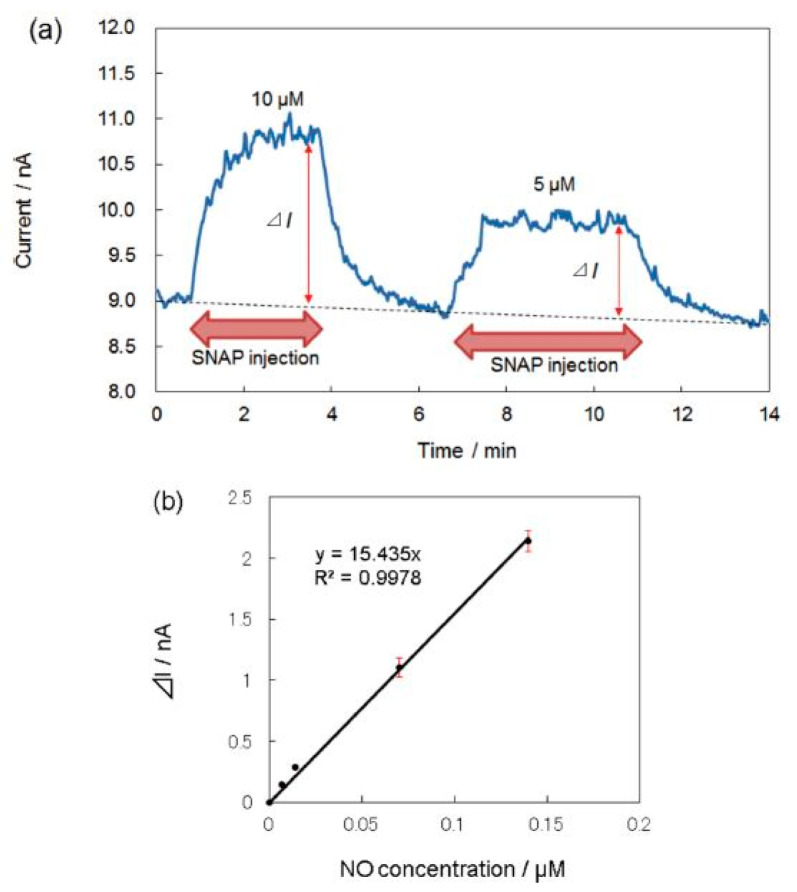
(**a**) Amperometric detection of NO at the FeT3ThP-modified electrode. The electrode potential was +0.8 vs. Ag/AgCl. (**b**) Calibration curve for NO detection at the FePor-modified electrode [58].

A layer of Nafion, which is a perfluorinated polymer network containing sulfonic acid groups, is deposited over the electroactive area often in order to eliminate interference of NO_2_^−^ and NO_3_^−^ forming during NO oxidation. The problems of interfering substances in the detection of NO were discussed in detail in the review [31]. Other common interfering compounds in biological systems are dopamine, acetaminophen, uric and ascorbic acids. For the determination of NO in the presence of the interfering compounds, various polymeric membranes were used, among them Nafion, polycarbazole, polystyrene, fluorinated xerogel and some others. It was shown that FeT3ThP-modified electrode detected NO species at +0.8 V vs. Ag/AgCl and exhibited the linear dependence on NO concentration in the range from 0.5 to 10 μM and the calculated LOD of 3 nM (Figure 5, Table 1).

**Figure 6:**
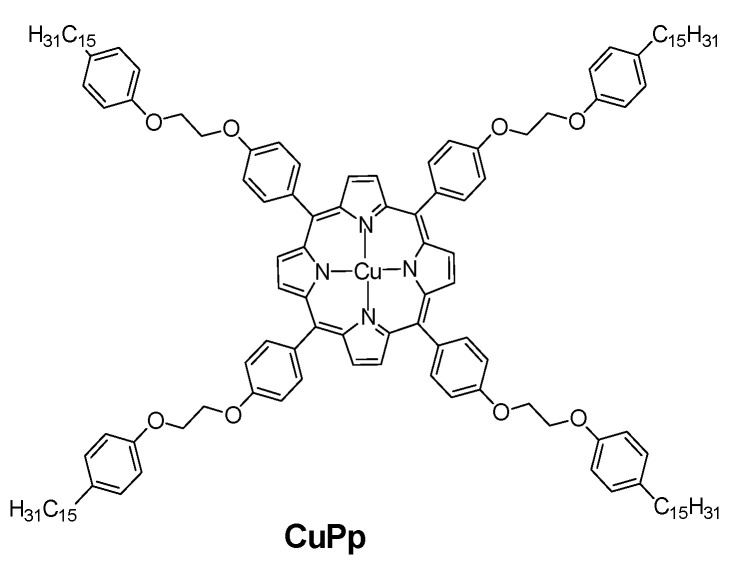
Structural formula of a Cu(II)Por complex used in Ref. [73] for the modification of Au electrode.

Oliveira et al. [73] modified the surface of the Au electrode with a Cu(II)Por complex (Figure 6) dissolved in dichloromethane in the presence of 0.1 mol·L^−1^ TBAP (tetrabutylammonium perchlorate) by CV in the potential range of 0–1.45 V. The anodic peak current was noticeably higher for the modified electrode, and its detection limit decreased to 6.18 × 10^−8^ M vs. 8.29 × 10^−7^ M for the unmodified Au electrode. The electrodes were also sensitive to dopamine, serotonin and nitrite. 

**Figure 7:**
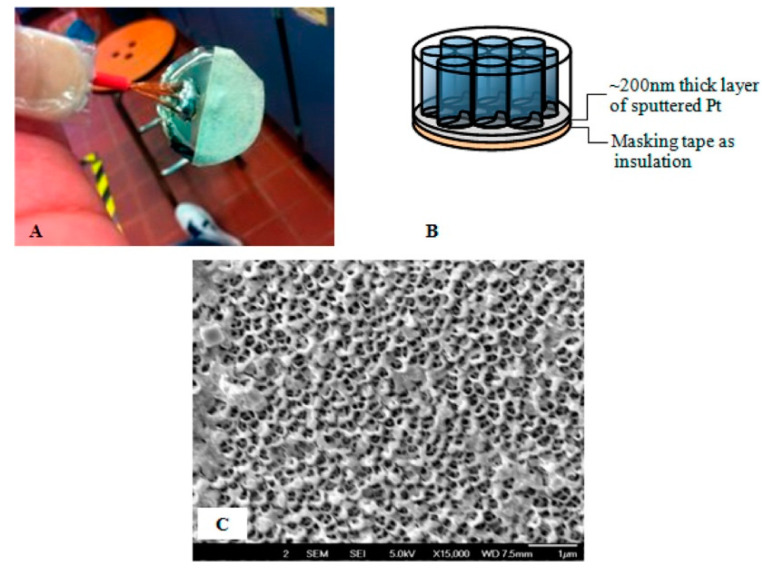
Photograph of the AAO/Pt electrode (**A**) with the general schematic shown in (**B**). FE-SEM of the AAO/Pt after dissolution of the AAO membrane filter shows the annular morphology of the sputtered Pt (**C**). Reprinted with permission from [74]. Copyright 2013 American Chemical Society.

Yap with co-authors [74] prepared electropolymerized 4′,4″,4‴,4⁗-tetraamine phthalocyanine (poly-MTAPc, M = Cu, Zn, Pt) modified electrodes for the detection of NO in water solutions. MTAPc complexes were electropolymerized both onto a glassy carbon (poly-MTAPc/GC) electrode and within the pores of a Pt-coated Anodisc nanoporous membrane (poly-MTAPc nanotube/AAO/Pt) (Figure 7). 

Due to its highly developed surface, the poly-MTAPc nanotube/AAO/Pt electrode demonstrated higher sensitivity to NO (4.44 vs. 0.0343 μA/μM·mm^2^ for CuTAPc) compared to the planar sample. Its detection limit was 10 nM, which was one order of magnitude higher compared to the flat electrode. In dependence on the central metal in phthalocyanine derivative, the sensitivity to NO increased in the order ZnTAPc (18 nA/nM) < CuTAPc (20 nA/nM) < PtTAPc (31.54 nA/nM).

The literature describes examples of the determination of NO by electrochemical methods in real biological samples. For example, Chandra et al. [59] reported that the modification of GCE by 5,10,15,20-tetrakis(4-methoxyphenyl) porphyrin (H_2_TMPP) allows the direct detection of NO in cultured cervical cancer (HeLa) cells. The three-electrode system including a Pt wire as counter electrode, silver reference electrode and GCE as working electrode was used for electrochemical NO detection. Firstly, authors investigated electrochemical performance of H_2_TMPP porphyrin in different media such as dichloromethane and phosphate buffer. Then electrochemical measurements of nitric oxide in cultured HeLa cells were provided using chronoamperometry. The NO sensor registered response (0.0138 nA/μL) of extracellular NO released upon activation of HeLa cells with a linear correlation coefficient.

Gonzalo Ramirez-Garcia et al. [75] demonstrated the direct detection of NO in rat kidneys using NiTSPc/polyphenol modified ultramicroelectrodes (a platinum wire 25 μm in diameter). The electrodes were covered electrochemically with a two-layered membrane consisting of nickel tetrasulfonated phthalocyanine (NiTSPc) and a selective polyphenol membrane. Detection limit of NO in water solutions of 25.4 nmol·L^−1^ was estimated for a signal/noise ratio of 3 and the linear range below 50 μM (Table 1). 

The direct detection of NO released from rat liver homogenate was also performed by Yong Li et al. [76]. They reported on the fluorescent NO biosensor, in which iron porphyrin of cytochrome P450 55B1 (CYP55B1) was utilized. It was shown that NO molecule bounded to iron porphyrin of CYP55B1, which led to a change in the fluorescence spectrum of cytochrome CYP55B1. The linear dependence of the response to NO was observed below 22.5 μM, and the detection limit was 0.15 μM (S/N = 3). The prepared NO biosensor exhibited high selectivity to NO in physiological solutions (rat liver homogenate).

### 2.2. Hybrids of Phthalocyanines and Porphyrins with Carbon Nanomaterials

The synergistic combination of the properties of porphyrinoids with those of carbon nanomaterials (viz. their one-dimensional electronic structure, high conductivity, large surface area) allows for obtaining NiPc and NiNc active layers with advanced sensor characteristics. Carbon nanomaterials were shown to improve the electron transfer rate when used for electrode modification, and even exhibited electrocatalytic properties in some electrochemical processes. On the other hand, the use of porous nanocarbon materials for modification of electrodes allows for increasing the area of their active surface. Application of hybrid materials with nanocarbon (CNT, graphene, rGO) provides a highly conductive bridge to facilitate rapid transport of electrons between the phthalocyanine and the electrode [80]. For the preparation of such hybrid materials, the methods of both non-covalent and covalent functionalization of carbon nanomaterials with phthalocyanines and porphyrins were used [80]. 

It is necessary to mention that the performance sensor is not only determined by the molecular characteristics, but also by the sensor design, which includes the choice of electrode material, the method of its modification, the introduction of additional binding components and membranes that ensure the selectivity of the sensor, and some others. For this reason, when analyzing the works available in the literature, attention was also paid to some of these aspects. Below we present examples of sensors based on such hybrid materials obtained over the past decade, focusing on methods for the preparation of such hybrid materials. The electrodes and the methods of their modification are indicated in Table 1.

**Figure 8:**
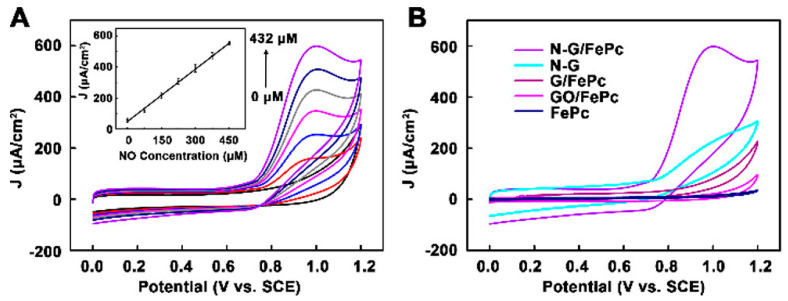
(**A**) CV of N-G/FePc/Nafion/PLL ITO electrode in 0.01 M PBS (pH 7.4) containing various concentrations of NO (0, 72, 144, 216, 288, 360, 432 μM). (**B**) CV of five different materials modified ITO electrode in 0.01 M PBS (pH 7.4) containing 432 μM NO. Reprinted with permission from [64]. Copyright 2018 American Chemical Society.

Huiying Xu et al. [64] created a novel sensing platform for in-situ monitoring of NO, which was based on nitrogen-doped graphene (N-G) nanocomposites modified with iron phthalocyanine via non-covalent interaction. An ITO electrode was modified with FePc/N-G hybrid material and then coated with Nafion and poly-L-lysine (PLL). The prepared FePc/N-G hybrid material was shown to demonstrate the best catalytic activity compared to the electrode modified with FePc and graphene oxide or graphene functionalized with FePc due to the combination of catalytic activity of FePc and N-G (Figure 8). 

**Figure 9:**
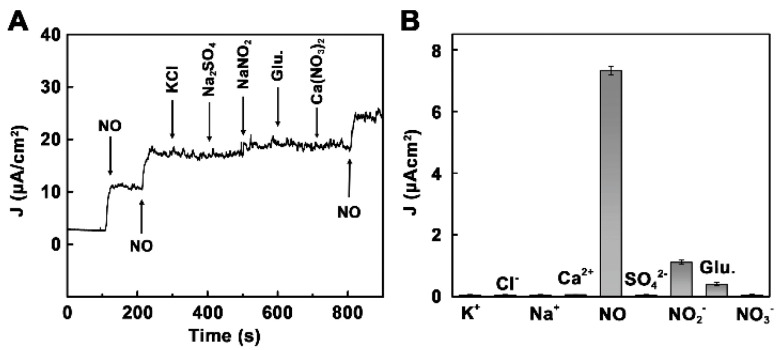
(**A**) Amperometric responses to 36 μM NO, KCl, Na_2_SO_4_, NaNO_2_, glucose and Ca(NO_3_)_2_. (**B**) The selective profile. Reprinted with permission from [64]. Copyright 2018 American Chemical Society.

The dependence of the response of N-G/FePc/Nafion/PLL ITO electrodes to NO was linear at the concentrations <432 μM in PBS (0.01 M) (Figure 8A), while the LOD was 180 nmol L^−1^ at a signal-to-noise ratio of 3. Due to the remarkable synergistic effect of the N-G and FePc nanocomposite layer, the biosensor displayed superior conductivity and excellent electrocatalytic activity toward NO oxidation. The N-G/FePc/Nafion/PLL ITO electrode demonstrated the larger current response to NO than to other investigated analytes (Figure 9), which indicated the possibility of NO detection in the presence of KCl, Na_2_SO_4_, glucose and Ca(NO_3_)_2_ in water solutions. Despite the use of a negatively charged Nafion film acting as a barrier to repel negatively charged nitrite due to electrostatic repulsion, a slight effect of NO_2_^−^ (signal changes <10%) was observed (Figure 9B). Apart from this, the researchers tested the possibility of the application of the N-G/FePc/Nafion/PLL ITO sensor for real-time and in situ monitoring of NO molecules released from Human Umbilical Vein Endothelial Cells (HUVEC).

**Figure 10:**
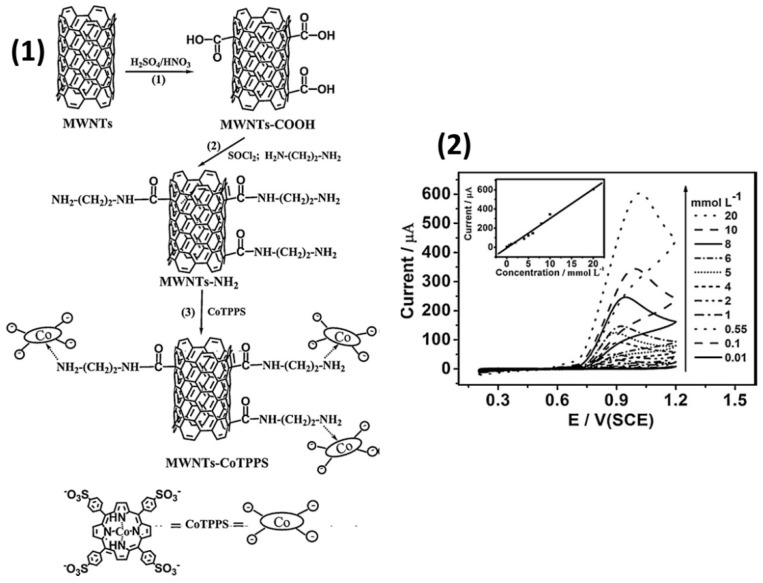
(**1**) Preparation process of the MWCNT-CoTPPS hybrid and the structure of CoTPPS. (**2**) Cyclic voltammograms of MWCNT-CoTPPS/GCE in PBS (pH 2.0) containing different concentrations of NaNO_2_ at a scan rate of 50 mV s^−1^ at 298 K. The inset shows the plot of peak currents vs. concentration of NaNO_2_. Adapted with permission from [55]. Copyright 2011 Elsevier.

Ya Yan et al. [55] modified the glassy carbon electrode with multi-walled carbon nanotubes (MWCNTs) functionalized with cobalt(II) tetrakis(4-sulfonatophenyl)porphyrin (CoTPPS). CoTPPS was attached on the surface of MWCNTs bearing -CO-NH-(CH_2_)_2_-NH_2_ via their axial coordination to Co cation (Figure 10(1)). Such types of coordination promote the stronger interaction between phthalocyanines and carbon nanotubes and help to prevent leaching of the electroactive component that was used to modify CNTs. The electrocatalytic activity of MWCNT-CoTPPS/GCE in the process of NO oxidation was investigated using cyclic voltammetry, impedance spectroscopy and chronoamperometry. NaNO_2_ was used as a source of NO because it hydrolyzes in acid solutions (pH < 4) to generate free NO.

It was shown that GCE modified with a MWCNT-CoTPPS hybrid exhibited larger anodic peak and as a result better electrocatalytic activity to NO oxidation than in the case of bare electrodes and GCE modified with MWCNT or CoTPPS separately. The electrode had high sensitivity to NO with the linear dependence of anodic peak current of NO on the concentration of NaNO_2_ in the range from 1 × 10^−5^ to 2 × 10^−2^ M (Figure 10 (2)). The calculated LOD was 6.6 × 10^−6^ M. 

Mukherjee and co-workers [77] reported the results of a study of an electrochemical sensor based on a hybrid material containing nitrogen-doped graphene nanosheets modified with 5,10,15,20-tetrakis(1-methyl-4-pyridino) porphyrin tetra(*p*-toluenesulfonate) by a low temperature hydrothermal method via non-covalent strategies. Due to its increased active surface area, which resulted in an increase in the number of reactive sites and low resistance, the prepared hybrid material had better sensitivity and selectivity toward nitric oxide compared to pristine graphene sheets and porphyrin. Its sensitivity to NO in water solution containing 0.1 M PBS (pH 7.4) was 3.6191 µA/µM with the calculated LOD of 1 nM. The authors presented in vitro studies of NO released from RAW 264.7 macrophage cells. The resulting hybrid material proved to be suitable for determining NO in real time.

**Figure 11:**
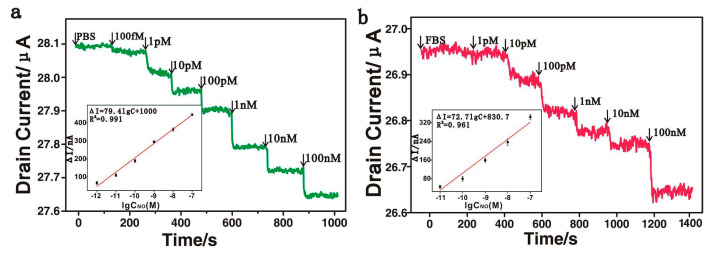
(**a**) Real-time electrical measurement at different concentrations of NO solution in PBS. Inset: the response of the rGO/FGPCs FET biosensors to NO at a series of concentrations (1, 10, 100 pM and 1, 10 and 100 nM) in PBS. (**b**) Real-time electrical measurement at different concentrations of NO solution in the cell medium. Inset: the response of the rGO/FGPCs FET biosensors to NO at a series of concentrations (10, 100 pM and 1, 10 and 100 nM) in fetal bovine serum. Reprinted with permission from [78]. Copyright 2016 American Chemical Society.

Another example of real-time monitoring of nitric oxide content was presented by Xie and co-authors [78]. The researchers prepared a field effect transistor (FET) based on reduced graphene oxide (rGO) non-covalently modified with Fe(III) meso-tetra(4-carboxyphenyl)porphyrin. The resulting hybrid was designated as an FGPC. In the FET device, rGO caused high electrical conductivity, and metal porphyrin provided highly catalytic activity. The combination of these properties led to a good sensitivity of the transistor to NO in the range from 100 fM to 100 nM with LOD of only 1 pM in PBS and 10 pM in human umbilical vein endothelial cell culture, respectively (Figure 11). This is the minimal LOD among the sensors summarized in Table 1. Selectivity was investigated by adding the same concentration (10 μM) of uric acid, sodium ascorbate, glycine, l-arginine, hydrogen peroxide and potassium nitrate. The sensor response to NO was about five times higher than to other analytes. In addition, the FGPCS-based sensor was shown to have excellent reproducibility, repeatability and stability.

Yan-Ling Liu et al. [81] also used the modified rGO for the development of a sensing array towards NO. To prepare the hybrid nanosheets (named FGHNs), rGO was non-covalently functionalized with Fe(III) meso-tetra(4-carboxyphenyl) porphyrin (FeTCP). The hybrid was deposited onto an ITO microelectrode array by electrophoretic deposition. The prepared nanosheets were functionalized covalently with 3-aminophenylboronic acid (APBA), which is typically used as cell-adhesive molecule because of its ability to react with the 1,2- or 1,3-diols of cell membranes [82]. The prepared FGHNs/ITO microelectrode array had higher sensitivity (37.6 μA·mM^−1^cm^−2^) to NO compared to that of rGO/ITO (7.2 μA·mM^−1^cm^−2^) and FeTCP/ITO (2.1 μA·mM^−1^cm^−2^) electrodes. APBA/FGHNs/ITO exhibited quite good selectivity to NO against many analytes like NO_2_^−^, ascorbic and uric acids, H_2_O_2_, L-arginine and acetylcholine, except dopamine and 5-hydroxy tryptamine. The APBA/FGHNs/ITO microelectrode was shown to be successfully used to detect NO released from human endothelial cells. Its sensitivity in the RPMI 1640 cell medium was about 70% of the sensitivity in the PBS solution, which was probably due to the adsorption of unwanted species of the cell medium onto the microelectrode surface. The response time was 600 ms in the cell medium, which was 1.5 time higher than in PBS.

**Figure 12:**
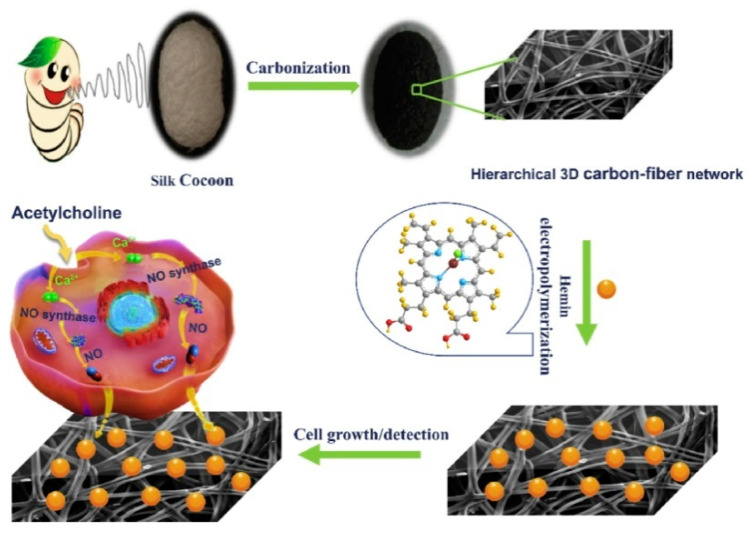
Illustration of preparation of the 3D hemin/CFN matrix and its cell adhesive and sensing application. Reprinted with permission from [53]. Copyright 2021 Elsevier.

It is also necessary to mention the work of Hu with co-authors [53] in which they use electrocatalytic properties of natural iron porphyrin of hemin to create a sensor for real-time monitoring of NO molecules in cancer and normal cells in live-cell assays. The 3D cell-adhesive sensing matrix was prepared on the basis of silk cocoon-derived hierarchical carbon fiber networks (CFN), on which iron porphyrin of hemin was assembled by the electrodeposition method (Figure 12). The prepared 3D hemin/CFN structure showed much better cell viability than other structured carbon materials. It was reported that the combination of hemin and CFN improved the electrical conductivity and catalytic activity towards NO. The obtained sensors demonstrated the excellent sensitivity to NO in a wide concentration range from 24.0 nM to 70.9 μM, fast response of 1.9 s and the detection limit as low as 8.0 nM in water solutions containing PBS (0.01 M, pH 7.4).

### 2.3. Hybrid Materials of Phthalocyanines and Porphyrins with MOF

It is well known that metal organic frameworks (MOF) combining the advantages of organic and inorganic parts are important materials with high porosity and large specific surface area and are attractive and useful for application as sensing materials [83]. The first examples of porphyrin-based MOFs [84,85] were described as early as the 1990s. Numerous efforts of researchers have been focused on studying the outstanding properties of these functional materials. In the case of phthalocyanine-based MOFs, fewer examples are available in the literature, but significant progress has been made recently. In recent years, the number of works on their use as active layers of chemiresistive, magnetic, ferroelectric, colorimetric and luminescent sensors has been growing rapidly [86,87,88]. It has been shown that hybrid materials including porphyrinoid molecules have better sensitivity and lower detection limits than pristine MOFs.

**Figure 13:**
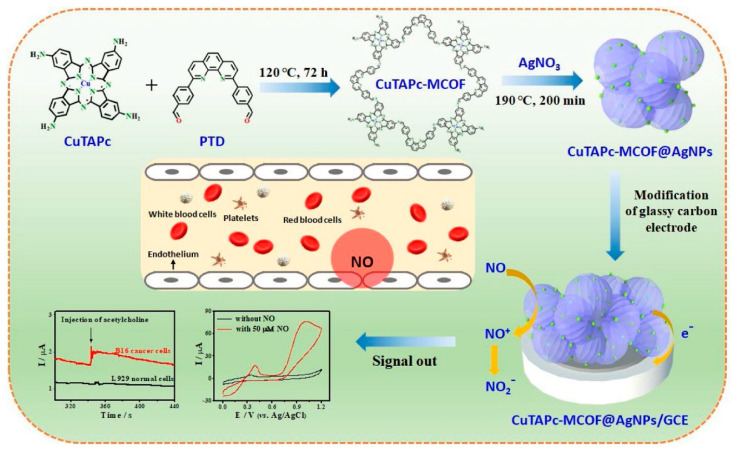
Schematic illustration of the synthesis of CuTAPc-MCOF@AgNPs nanohybrid for electrochemical detection of NO released from living cells. Reprinted with permission from [54]. Copyright 2021 Elsevier.

M. Wang with co-authors [54] prepared a new CuPc-based covalent-organic framework (CuTAPc-MCOF) doped with Ag nanoparticles (CuTAPc-MCOF@AgNPs). To synthesize CuTAPc-MCOF, the researchers used the Schiff base condensation reaction between Cu(II) 4′,4″,4‴,4⁗-tetra-aminophthalocyanine (CuTAPc) and 2,9-bis[*p*-(formyl)phenyl]-1,10-phenanthroline (Figure 13). The prepared porous material had the BET-specific surface area of 150.7 m^2^/g and showed a narrow pore size distribution of about 3.6 nm and a wide distribution in the range of mesopores and macropores. Its electrochemical response to NO was studied by a CV technique. The embedded AgNPs were used to increase the electrocatalytic ability and to improve the biocompatibility of the hybrid material. CuTAPc-MCOF@AgNPs/GCE exhibited the highest peak current of 76.4 μA toward NO oxidation, which was 2.1 times larger than that of CuTAPc (36.3 μA) and 1.3 times larger than that of CuTAPc-MCOF (59.3 μA). CuTAPc-MCOF@AgNPs had the better sensor performance compared to CuTAPc-MCOF and CuTAPc, with a sensitivity to NO of 29.1 μA·μM^−1^ cm^−2^, LOD of 12.6 nM and linear range from 0.18 to 17.1 μM (Table 1).

**Figure 14:**
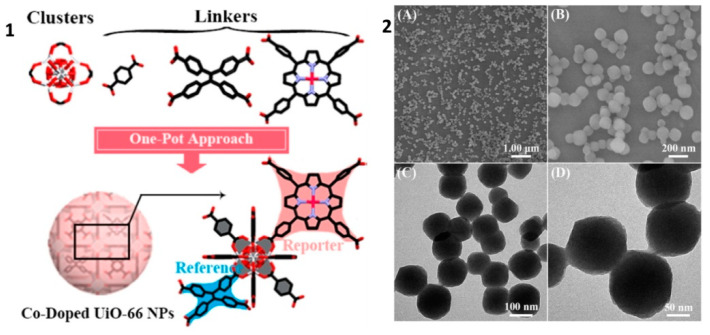
(**1**) Picture illustration for the synthesis of Pt-TCPP/H4TCPE@UiO-66NPs using one-pot in situ synthetic strategy. (**2**) (**A**) FESEM and the corresponding (**B**) magnified images, (**C**) TEM and the corresponding (**D**) magnified images of the Pt-TCPP/H_4_TCPE@UiO-66 NPs. The doping ratios of Pt-TCPP and H_4_TCPE to BDC in the NPs were set as 0.093:1 and 0.008:1, respectively. Reprinted with permission from [79]. Copyright 2019 Elsevier.

Ye Y. and co-workers [79] prepared a nitric oxide sensing material based on Zr-containing MOF UiO-66. Platinum meso-tetra(4-carboxyphenyl)porphyrin (Pt-TCPP) were integrated in the MOF structure through an in situ one-pot synthetic approach, in which 1,4-dicarboxybenzene (BDC) and/or 1,1,2,2-Tetra(4-carboxylphenyl)ethylene (H_4_TCPE) were co-linked with Zr-O clusters together (Figure 14). In the investigated hybrid material, the porphyrin complex played the role of an active site for the determination of nitric oxide, while H_4_TCPE worked as a luminescence reference and UiO-66 MOF prevented dye quenching effects caused by their aggregation and poor dispersibility in water.

**Figure 15:**
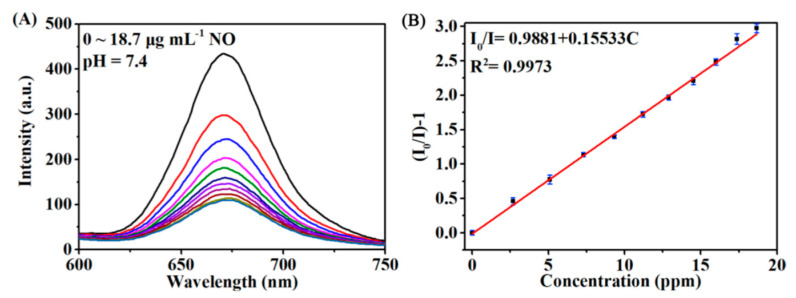
(**A**) Evolvement of the luminescence spectra of the Pt-TCPP/H4TCPE@UiO-66 NPs as a function of NO concentration from 0 to 18.7 μg mL^−1^ at pH = 7.4. (**B**) The corresponding Stern-Volmer plots of the quenching luminescence intensity as a function of NO concentration at pH = 7.4. Reprinted with permission from [79]. Copyright 2019 Elsevier.

For the NO detection, the luminescent spectra with an emission wavelength at 670 nm in HEPES buffer solution were recorded before and after injection of different concentration of analyte. The spectra were studied at different pH values (0, 5.6, 7.4). The studied sensing platform exhibited a good linear dependence on the NO concentration from 0.48 to 18.7 µg·mL^−1^, a fairly fast response time (2 min) and a low detection limit of 0.142 µg·mL^−1^ at pH = 7.4 (Figure 15).

Selectivity of Pt-TCPP/H_4_TCPE@UiO-66 nanoparticles was studied in the presence of ClO^−^, NO_3_^−^, ONOO^−^ and H_2_O_2_ species. The response toward nitric oxide was found to be higher than toward other analytes. Taking into account the UV-vis absorption spectra and the emission decay data, the mechanism of the NO-detection could be ascribed to a static quenching. Pt-TCPP/H_4_TCPE@UiO-66 NPs had good biocompatibility and was tested as a material for real-time NO sensing in HeLa living cells. 

The literature analysis for the past 10 years shows that metal phthalocyanines, porphyrins and their hybrid materials are mostly used as active layers of electrochemical sensors for the detection of nitric oxide in water media, although there are several examples of their use in fluorescent ones. When designing sensitive and selective NO electrochemical sensors based on metallic phthalocyanines and porphyrins, attention should be paid not only to the preparation of the sensing material itself, but also to the choice of electrode materials, the manner of its modification with sensing material and the introduction of membranes that ensure the selectivity of the sensor. An analysis of the literature shows that phthalocyanines and porphyrins of electroactive metals such as cobalt and iron are most often used to modify sensitive electrodes for electrochemical detection. At the same time, the effect of the type of substituents in the aromatic ring on the electrocatalytic properties of these molecules and their hybrid materials has not been sufficiently investigated, and the choice of one or another substituent is usually not justified in the works.

Along with the use of MPc and MPor themselves for the modification of electrodes, the attention of researchers has been directed to the study of their hybrid structures with carbon nanomaterials and MOFs in order to increase the active surface area and sensor sensitivity (Table 1). Improved sensor sensitivity is also achieved by increasing the surface area of the electrode. For this purpose, along with polishing and activation of classical conductive electrodes, porous membranes coated with metals are used [74]. An important step in the development of the sensor is also the method of modification of the electrode surface, which provides good adhesion and charge transfer between the active layer and the electrode surface. The methods, which are widely used for the modification of electrodes with phthalocyanine, porphyrins and hybrid materials, include immersion in the corresponding solution [56], drop and dry [59,64,77,78], electrodeposition or electropolymerization [58,73,74]. To prevent leaching of the electroactive component, the active layers are sometimes covered with a polymeric layer that does not prevent the penetration of analyte molecules. All these methods have their own advantages and limitations and are determined by the properties of sensing materials, as well as by analytical tasks.

A useful strategy aimed at improving the selectivity of the sensor to NO involves the use of various polymeric membranes, e.g., Nafion, which provide a sufficient barrier for various anionic interfering analytes, especially nitrite [59,61]. Indeed, the chemical modification of electrode surfaces with metal phthalocyanine and porphyrin films, polymer and polyelectrolytes expands the scope of application of such new-design electrodes and provides many opportunities for their use in various experimental conditions.

## 3. Sensing Layers for the Detection of Gaseous NO

Literature analysis shows that apart from commercially available chemiluminescence instrument [89], optical [90], electrical [91] and QCM [92] techniques are used for the detection of gaseous NO and FENO. The examples of phthalocyanine and porphyrin-based sensors used for the detection of gaseous NO are presented in Table 2. In the sensors with electrical response, in which the response is associated with a change in the conductivity of the active layer in the atmosphere of a gaseous analyte, the active layer can be deposited onto interdigitated electrodes or field effect transistor structures. For the deposition of phthalocyanine and porphyrin films the methods of vacuum evaporation, spin coating, drop casting are usually used. 

The nature of interaction between the active layer and the NO molecule is still the subject of discussion in different research papers. To explain the mechanism of sensor response, two theories are most often described in the literature. The first mechanism is based on the displacement of adsorbed oxygen molecules from the surface of a metal phthalocyanine or porphyrin film [93]. On the sensing active layer surface charge-transfer complexes MPc···O_2_ (MPc^+^ and O^2−^ species) are formed. When a semiconductor gas sensor based on metal phthalocyanines and porphyrins is exposed to NO, the electrons injected into the material through the oxidation reaction between the reducing gas and the O^2−^ species on the semiconductor surface lead to a change in the film conductivity. In the second theory, a direct coordination of nitric oxide molecules to the central metal ion of MPc or MPor takes place [68,69]. Exposure of MPc and MPor films or their hybrid materials to gaseous nitric oxide results in a change in conductivity due to the depletion of positively charged holes by electrons donated by NO molecule.

An analysis of the literature shows that when determining gaseous NO, both methods of its direct determination and the determination of its oxidation product, namely NO_2_, are used. Due to the low stability of NO in air, its determination is possible only with strict control of the inert atmosphere. To do this, researchers use ultrahigh purity inert gases as diluent and purging gases and a sealed gas cell, usually made of stainless steel.

To eliminate the problems associated with the oxidation of NO to NO_2_ during measurements, some researchers first quantitatively oxidize NO to NO_2_, and then conduct a study of the sensor response to NO_2_ [90]. For the quantitative conversion of NO to NO_2_, NO is passed through a special cell containing oxidizing agents. Magori et al. [94] suggested utilizing the portable device that was based on a field effect transistor with a suspended gate and CuPc as a semiconducting layer for FENO measurement. For a more accurate measurement of FENO in exhaled air, NO was quantitatively oxidized to NO_2_. This was achieved by passing a gas mixture containing NO through a cell filled with porous silica gel to remove excess moisture and potassium permanganate (KMnO_4_) suspended on silica gel to oxidize NO to NO_2_. The sensor response was tested in the range of NO concentrations from 10 to 200 ppb and at the temperature of 95 °C. The device was shown to be used for selective detection of NO_2_ in the presence of CO, acetone, ethanol and ammonia. The authors demonstrated its application for FeNO measurements with artificial breath and real person probes.

**Figure 16:**
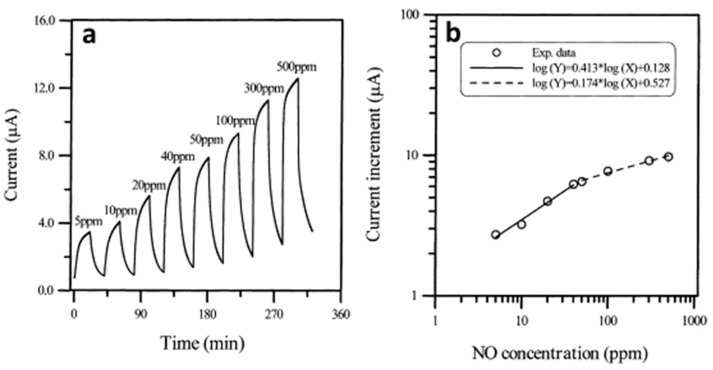
(**a**) Response current of NiPc film sequentially exposed to 5, 10, 20, 40, 50, 100, 300, and 500 ppm NO in N_2_ at 160 °C. Film thickness: 100 nm, substrate temperature: 25 °C, evaporation rate: 0.1 nm/s, mass flow rate 200 mL/min, adsorption time: 20 min, desorption time: 20 min without aerating N_2_. (**b**) All-logarithmic plot of current increase vs. NO concentration. Adapted with permission from [93]. Copyright 2001 Elsevier.

K.C. Ho et al. [93] produced chemiresistive sensors for the detection of gaseous NO, in which NiPc films were used as active layers. α-phase NiPc films (100 nm) were deposited by thermal evaporation in vacuum onto an Al_2_O_3_ substrate with pre-deposited interdigitated Au electrodes at the substrate temperature 25 °C. Ultrahigh purity N_2_ was used as a diluting and purging gas. The electrical conductivity of NiPc films increased upon interaction with NO (Figure 16).

The sensors were not completely reversible. To reach the steady state more than 500 mins were required. In the NO concentration range from 5 and 50 ppm, the sensitivity was between 0.41 and 0.42, while in the range from 50 and 500 ppm, the sensitivity decreased to about 0.17 to 0.19. These facts may indicate that unstable NO transformed into NO_2_ during the measurement, and the researchers measured the sensor response to NO_2_. The same group of authors [100] studied ethanol-treated PbPc films as active layers of sensors to NO vapors and compared their sensitivity to NO with that of as-deposited films. They found that the sensitivity of ethanol-treated PbPc films was 0.65, while in the case of as-deposited films the sensitivity was only 0.38. The authors attributed this noticeable increase in sensitivity to a change in the morphology of the films, namely an increase in grain size.

**Figure 17:**
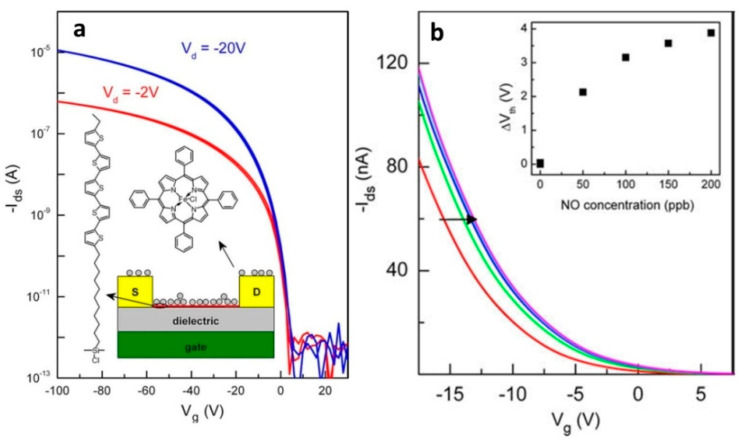
(**a**) Transfer characteristics of a typical SAMFET in vacuum with a 1 μm SiO_2_ gate dielectric in the linear and saturated regime. The device exhibited p-type behavior with a pinch off voltage around 0 V. The inset shows a schematic cross-section of the SAMFET sensor. The chemical structures of the SAM molecule (left) and the NO receptor Fe(TPP)Cl (above) are shown. (**b**) Linear plot of the transfer characteristics of the SAMFET sensor. The measurements performed in vacuum and nitrogen were identical. The transfer curve measured 30 min after exposure and shifted towards positive values for increasing NO concentrations. The inset shows the threshold voltage shift as a function of NO concentration. Adapted with permission from [95]. Copyright 2010 Elsevier.

Andringa et al. [95] developed a sensitive gas sensor based on a self-assembled monolayer field-effect transistor (SAMFET) where iron(III) tetraphenylporphyrin chloride (Fe(TPP)Cl) was used as a specific receptor to detect NO (Figure 17a). 

The response of the SAMFETs was measured by admitting small amounts of NO diluted in N_2_ carrier gas. The measurements were carried out in both vacuum and nitrogen. It was demonstrated that the transfer curve systematically shifted to positive gate biases with an increase in the NO content (Figure 17b). The LOD was found to be 100 ppb. The selectivity of the SAMFET sensor was also investigated toward both oxidizing and non-oxidizing gases. No or negligible threshold voltage shift was observed for toluene (8 ppm), methanol (%), ammonia (2 ppm) and O_2_ and SO_2_ gases. Reversibility of the sensor after NO detection was also examined. Under vacuum conditions at 110 °C for 1 h, the threshold voltage completely returned to its original value. 

**Figure 18:**
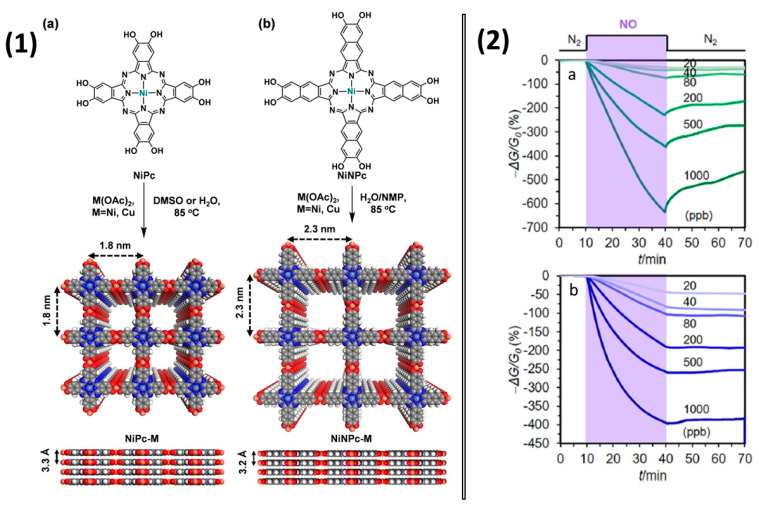
(**1**) Synthetic scheme for isoreticular phthalocyanine and naphthalocyanine-based MOFs (**a**) NiPc-M and (**b**) NiNPc-M. Top and side view of their structures with 2 × 2 square grids in eclipsed stacking mode. (**2**) Response of NiPc-Ni MOF (**a**) and NiPc-Cu MOF (**b**) to NO at different concentrations. Reprinted with permission from [96] Copyright 2019 American Chemical Society.

Interesting work was done by Z. Meng et al. [96], in which they prepared 2D meshes on the basis of phthalocyanine and naphthalocyanine bimetallic 2D metal-organic frameworks (MOFs) NiPc-M and NiNPc-M (M = Ni, Cu) (Figure 18(1)). The prepared MOFs had porous structure with BET surface areas of 101−284 m^2^/g. The framework structures based on NiPc and NiNc allowed for improving the conductivity of the material by five–seven orders of magnitude compared to the initial phthalocyanines. The active layers with a thickness of 1–6 μm were obtained by drop casting of 10 μL of MOF suspension (1–4 mg/mL in H_2_O) onto IDE (5 μm gap) and testing with different NO concentrations (Figure 18(2)).

NiPc-Ni and NiPc-Cu showed current changes of −657% and −397%, respectively, after 30-min exposure to 1 ppm NO. They demonstrated a negative response when exposed to NO, suggesting that NO acted as an electron acceptor when interacting with the investigated frameworks. The linear dependence of the response on the concentration of NO was observed in the range of 0.02–1.0 ppm of NO, while the calculated LOD was 1.0–1.1 ppb for a 1.5-min exposure. 

In addition to chemical sensors with electrical response, quartz crystal microbalance sensors in which metal porphyrins and phthalocyanines are active layers were also used for NO detection. For instance, W. Tao and co-authors [97] developed a QCM sensor in which a CoPc-silica hybrid material was utilized for the detection of gaseous NO. The CoPc-silica hybrid was prepared by sonication of suspension of SiO_2_ and solution of CoPc in dimethylsulfoxide for 1 h. Nitrogen was used as a diluting and purging gas. The sensing mechanism was based on the coordination adsorption of NO molecules on CoPc, which led to a resonant frequency shift of the modified QCM sensor. A linear range for NO was from 5.75 to 103.45 ppb, and the detectable NO concentration was 5.75 ppb. 

**Figure 19:**
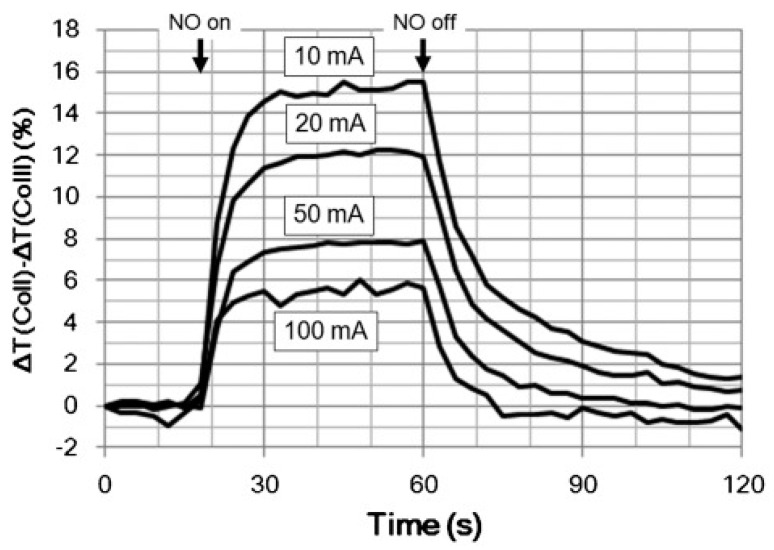
Transient sensor response changes during exposure (1 ppm NO) and recovery (N_2_) for the Co-TPP/cellulose sensor measured at different LED driving currents. Reprinted with permission from [90]. Copyright 2016 Elsevier.

Tetraphenylporphyrins (TPP) are known to be promising chemochromic materials [101]. Miki et al. [90] developed a tetraphenylporphyrin-based optical sensor with excellent sensitivity for detecting NO with an accuracy of ±1 ppb. The sensors were manufactured by embedding CoTPP derivatives into porous nonwoven materials (viz. cellulose). The use of nonwoven fabrics reduced the response time to 1 s for 10 ppb of nitric oxide. To improve the reversibility of the response the sensor was heated and illuminated by light from the LED simultaneously (Figure 19). The researchers demonstrated that the CoTPP-based sensor was promising for detecting NO in human breath because it had good selectivity to NO in the presence of CO_2_, H_2_S, NH_3_ and CO. They developed a prototype analyzer using volunteers’ breath samples with a detection limit below 1 ppb and a response time of 1 s. Nevertheless, the response of the developed sensor fluctuated significantly depending on the change in humidity.

**Figure 20:**
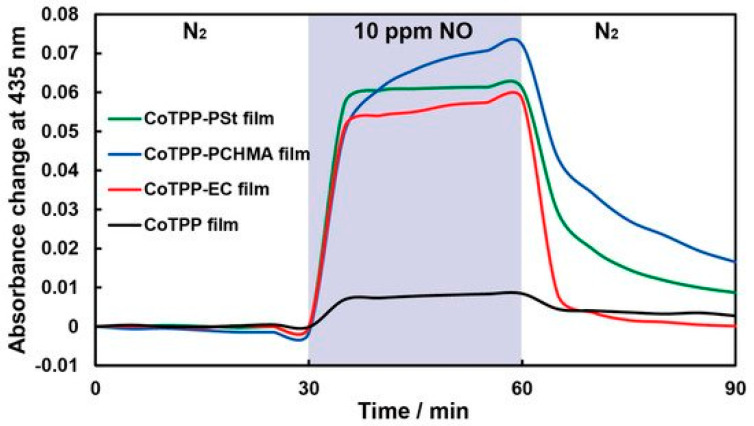
Typical responses of CoTPP film (black), CoTPP-EC film (red), CoTPP-PCHMA film (green) and CoTPP-PSt film to 10 ppm of NO/N_2_ at 100 °C. The blue zone indicates the period of NO gas introduction [98].

To reduce the sensitivity to moisture, researchers often use hydrophobic polymers, both for deposition on top of a sensitive film and for obtaining composite materials. Shiba et al. [98] demonstrated how CoTPP dispersed in three hydrophobic polymer matrixes (polystyrene, ethylcellulose (EC) and polycyclohexyl methacrylate) affected the moisture resistance of the optical sensor to NO. The presence of a hydrophobic polymer matrix led not only to an increase in stability in a humid atmosphere, but also to an increase in sensitivity by more than six times due to the suppression of CoTPP aggregation. As shown in Figure 20, CoTPP/polymer films exhibited seven-eight times greater response to 10 ppm of NO gas than CoTPP film, which indicated that NO gas could penetrate the polymer and change the absorbance spectra of CoTPP molecules. The limit of NO detection was 33 ppb in the case of CoTPP-EC film, which was lower than for a CoTPP film (92 ppb).

**Figure 21:**
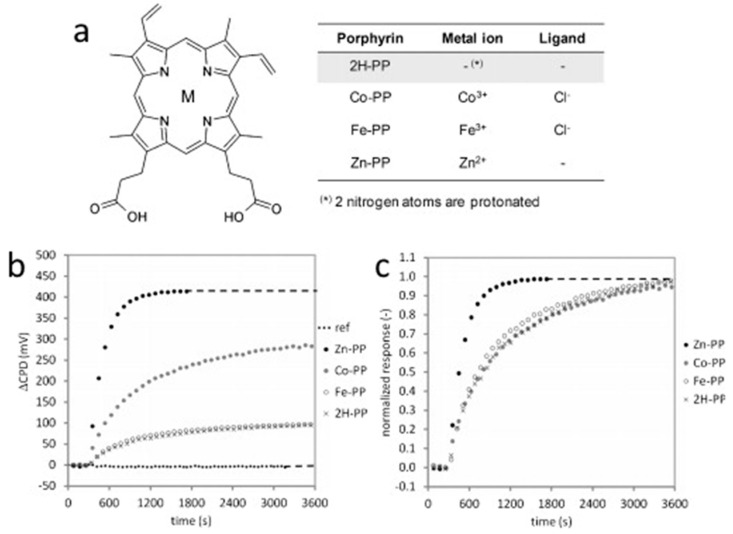
(**a**) Molecular structure of the used protoporphyrins IX. M indicates the position of the metal ion. (**b**) Kelvin probe response of the different porphyrins to 2 ppm NO; (**c**) same as (**a**), normalized to the equilibrium response. Adapted with permission from [99]. Copyright 2012 Elsevier.

The chemistry of natural porphyrins, like protoporphyrins IX, and cytochromes is currently of great interest and is also used in the development of sensor devices. One potential application is the detection of gaseous NO. Knoben et al. [99] investigated the sensor properties of four different protoporphyrins IX (PP, Figure 21a) adsorbed on the surface of native alumina oxide formed on 100 nm Al film sputtered on a Si wafer by a Kelvin probe technique. The monolayers of 2H-PP, Co-PP, Fe-PP and Zn-PP were prepared by immersion of the substrate to their solutions in dimethylformamide overnight. The concentration of NO in the flow cell was varied from 100 ppb to 2 ppm. The Kelvin probe response, which is a contact potential difference (CPD), is shown in Figure 21b,c for four different protoporphyrins. It was found that Zn-PP exhibited the largest and the fastest response to NO and allowed for determining NO at the ppb level in environmental conditions.

The authors also investigated mixed monolayers Zn-PP/FBA in which 4-fluorobenzoic acid (FBA) was used as a spacer molecule due to strong π–π stacking with the protoporphyrin molecules. The mixed monolayers showed the lower sensor response to NO, but the response rate was higher than in the case of Zn-PP monolayer. 

The analysis of the last works shows that metal phthalocyanines, porphyrins and their hybrid materials are mostly used as active layers of chemiresistive, optical and QCM sensors for the detection of gaseous NO and FENO. MPc and MPor films deposited by spin coating and PVD methods continue to be used for the preparation of active layers of these sensors [93,95,99]. However, along with them, researchers create new materials such as 2D MOFs on the basis on phthalocyanine and naphthalocyanines [96], as well as hybrid materials with nanoparticles [97], which have great potential due to their porous structure, large surface-to-volume ratio, and comparatively simple preparation techniques.

## 4. Advances, Current Issues and Future Scope 

This review provided an overview of the state of research over the past ten years in the field of sensors based on porphyrins, phthalocyanines and their hybrid materials for NO detection both in aqueous solutions and in the gas phase. The current scenario of the development of sensors for the determination of NO in aqueous solutions and biological media is focused on the search for new materials, which can increase of their sensitivity and selectivity as well as to provide a possibility of their use in real biological media. Along with the use of phthalocyanines and porphyrins themselves for the modification of electrodes, in the last decade the attention of researchers has been directed to the study of their hybrid structures with carbon nanotubes, graphene, ordered mesoporous carbon, etc. The synergistic combination of the properties of porphyrinoids with those of carbon nanomaterials (viz. their one-dimensional electronic structure, high conductivity, large surface area) allows us to facilitate rapid transport of electrons between the aromatic macrocycle and the electrode and obtain the active layers with advanced sensor characteristics. Other promising materials for the electrodes’ modification can be obtained by incorporation of porphyrins and phthalocyanines into metal-organic frameworks due to their structural characteristics such as high porosity and high specific surface area. Currently, such materials are not yet as well studied as hybrid materials with nanocarbon, although it is already becoming clear that these materials may have a great future due to their large surface-to-volume ratio, which can lead to the formation of a porous conductive interface with a high surface area that can increase the adsorption capacity of the surface and provide sensitive detection of NO. Apart from this, the wide variety of MOFs and the possibility of their chemical tailoring allow for adapting these materials to different types of electrode surface to improve the analytical characteristics of nitric oxide sensors. It is also important to note that hybrid materials based on porphyrinoids with carbon nanomaterials and MOFs can be used not only for the determination of nitric oxide in aqueous solutions, but also in chemiresistive sensors for the detection of gaseous NO.

Although there are already a number of indications in the literature on the unique properties of phthalocyanines, porphyrins and their hybrid materials in NO sensors, in our opinion, further research in this field should be focused on (i) the search for new ways of synergistic combination of porphyrins and phthalocyanines with nanostructured materials and/or ordered structures; (ii) the development of new materials stable for a long time in various buffered salt solutions and biological media and capable of specific binding for the manufacture of nitric oxide sensors with better selectivity; (iii) the search for materials for the creation of multisensor systems and arrays both for environmental monitoring and for medical applications; (iv) the creation of biocompatible and miniature systems for the observation and determination of NO in vivo. 

## Figures and Tables

**Table 1:** Examples of phthalocyanine and porphyrin-based sensors used for the detection of NO in aqueous solutions and biological media.

Sensing Layer	Method/Electrode	Sensing Layer Preparation/Deposition	Analyte	Linear Range, μM	LOD, μM	Ref.
1,8,15,22-tetraamino- phthalocyanatocobalt(II) (4α-Co^II^TAPc)	Amperometry, DPV/GCE	Immersion of the electrode in 4α-Co^II^TAPc solution in DMF	NO in PBS (0.2 M, pH 2)	0.003–0.03	1.4 × 10^−4^	[56]
Fe tetrakis(3-thienyl)porphy- rin (FeT3ThP)	Amperometry, DPV/GCE	Electropolymerization/coating with Nafion	NO in PBS (0.15 M)	0.5–10	0.003	[58]
Cu(II)Por complexes (Figure 5)	CV/Au electrode	Electropolymerization	NO, aqueous solution with electrolytes (Na_2_SO_4_, TBAP)	0.282–2.85	0.0618	[73]
Pt 4′, 4″, 4‴, 4′′′′-tetraamine phthalocyanine	Pt-coated Anodisc nanoporous membrane	Electropolymerization/coating with Nafion	NO in PBS (pH 7.4)	0.01–0.1	0.01	[74]
5,10,15,20-tetrakis(4-me- thoxyphenyl)porphyrin (H_2_TMPP)	Amperometry/GCE	Drop casting	NO released from HeLa cells	-	1 × 10^−4^	[59]
Nickel tetrasulfonated phthalocyanine (NiTSPc)	CV/Ultramicroelectrode (25 μm Pt wire)	Electropolymerization/coating with polyphenol	NO released from HeLa cells	<50	0.0254	[75]
Iron porphyrin of cytochrome P450 55B1	Fluorescence spectroscopy	-	NO in rat liver homogenate	<22.4	0.15	[76]
N-G/FePc	Amperometry/ITO electrode	Non-covalent functionalization of N-G/drop casting	NO in PBS (0.01 M, pH 7.4)	0.18−400	0.18	[77]
MWCNT/Co(II) tetrakis(4-sulfonatophenyl)por-phyrin (CoTPPS)	Amperometry/GCE	CoTPPS was attached on MWCNT bearing -CO-NH-(CH_2_)_2_-NH_2_ via their axial coordination to Co/drop casting	NaNO_2_ in PBS (pH 2.0) was used as a source of NO	10–20,000	6.6	[55]
N-doped graphene nanosheets (PFNGS)/5,10,15,20-tetra- kis(1-methyl-4-pyridino) porphyrin tetra (*p*-toluenesulfonate)	Amperometry/Pt electrode	Non-covalent functionalization of PFNGS/drop casting	NO in PBS	0.001–10	0.001	[77]
rGO/Fe(III) meso-tetra(4-carboxyphenyl)porphyrin	Field-effect transistor	Non-covalent functionalization of rGO/drop casting	NO in PBS NO in Human Umbilical Vein Endothelial cells	-	1 × 10^−6^ 1 × 10^−5^	[78]
Iron porphyrin of hemin/CFN	Amperometry/carbon fiber networks	Electrodeposition	NO in PBS (pH 7.4)	0.024–70.9	0.008	[53]
Cu(II) 4′,4″,4‴,4⁗-tetra-amino-phthalocyanine/MCOF@AgNPs	Amperometry/GCE	CuPc-based covalent-organic framework doped with Ag nanoparticles/drop casting	NO in PBS (pH 7.4)	0.18–17.1	0.0126	[54]
Pt meso-tetra(4-carboxyphenyl)porphyrin/UiO-66	Luminescence spectroscopy	Pt-TCPP and H4TCPE integrated in MOFs UiO-66	NO in HEPES buffer (pH 7.4)	16–623.3	4.73	[79]

**Table 2:** Examples of phthalocyanine and porphyrin-based sensors used for the detection of gaseous NO.

Sensing Layer	Method	Linear Range, ppb	LOD, ppb	Ref.
Co(II) tetraphenylporphyrin (CoTPP)/porous nonwoven materials	Optical absorption spectroscopy	-	1	[90]
α-phase NiPc films (100 nm)	Chemiresistive	5000–50,000	-	[93]
Fe(III) tetraphenylporphyrin chloride (Fe(TPP)Cl)	Self-assembled monolayer field-effect transistor (SAMFET)	-	100	[95]
Phthalocyanine and naphthalocyanine bimetallic 2D MOFs NiPc-M and NiNPc-M (M = Ni, Cu) (Figure 18)	Chemiresistive	20–1000	1.0–1.1 (for 1.5 min exposure)	[96]
CoPc-silica hybrid material	QCM	5.75–103.45	5.75	[97]
Co(II) tetraphenylporphyrin (CoTPP)/polymer (polystyrene (PSt), ethylcellulose (EC), polycyclohexyl methacrylate (PCHMA))	Optical absorption spectroscopy	100–1000	33 (for CoTPP dispersed in EC)	[98]
2H-PP, Co-PP, Fe-PP and Zn-PP on Al_2_O_3_	Kelvin probe technique	100–2000	5	[99]

## Data Availability

Sharing data is not applicable.
